# Assessment of Hyoid Bone Movement by Simultaneous Ultrasound and Videofluoroscopic Imaging

**DOI:** 10.1007/s00455-026-10929-4

**Published:** 2026-03-24

**Authors:** Nicholas S. Schoenleb, Brittany N. Krekeler, Anna K. Hopkins, Suzanne E. Boyce, Rebecca J. Howell, David W. Rule, T. Douglas Mast

**Affiliations:** 1 Department of Biomedical Engineering, University of Cincinnati, Cincinnati, Ohio, USA; 2 Department of Otolaryngology-Head and Neck Surgery, University of Cincinnati, Cincinnati, Ohio, USA; 3 Department of Communication Sciences and Disorders, University of Cincinnati, Cincinnati, Ohio, USA; 4 Northside Hospital, Decatur, Georgia, USA

**Keywords:** Swallowing, Dysphagia, Hyoid motion, Modified barium swallow study (MBSS), Simultaneous ultrasound and videofluoroscopy, Transducer motion correction

## Abstract

Ultrasound imaging shows promise for bedside tracking of oropharyngeal swallowing structures, but has not been validated against videofluoroscopy. The hyoid bone is readily visible on both ultrasound and fluoroscopy and has multiple muscular attachments involved in execution of swallowing movements. This study aims to validate ultrasound measurements of hyoid motion by quantitatively comparing simultaneously recorded ultrasound and videofluoroscopy data. Participants (*N* = 23) were recruited from standard-of-care videofluoroscopy studies during which an ultrasound array was positioned submentally to acquire simultaneous B-mode ultrasound imaging. Kinematic analyses of hyoid motion in both ultrasound and videofluoroscopic image data from 56 swallows were analyzed to validate the accuracy of ultrasound measurements. Methods for correction of transducer movement included static angle correction, based on the initial transducer angle, and dynamic translation/rotation, based on the time-dependent transducer position. Hyoid motion variables were compared using intra-class correlation coefficients, root-mean-square error, *t* tests, and *F* tests, and included time-dependent trajectories, maximum excursion, and peak velocity. Ultrasound measurements were corrected using dynamic translation/rotation closely agreed with videofluoroscopy (ICC = 0.892 for hyoid trajectories; mean ICC = 0.715 for summary statistics). A substantial portion of hyoid position measurement error was due to transducer motion (estimated as 0.37 cm). These results indicate that ultrasound imaging, when incorporating appropriate correction for transducer motion, can accurately track motion of the hyoid bone and serve as a useful technique for measuring swallowing movements.

## Introduction

Abnormal swallowing (dysphagia) affects individuals across the lifespan and is particularly prevalent with increasing age. Dysphagia affects over 50% of long-term care residents and 30% of community-dwelling elderly [1, 2]. Consequences of dysphagia vary, but range from socio-emotional impact, including isolation and depression, to more life-threatening consequences including aspiration pneumonia, malnutrition and dehydration [3, 4]. The gold-standard dysphagia diagnostic approach for evaluating the oral, pharyngeal, and esophageal phases of swallowing is x-ray videofluoroscopy via the modified barium swallow study (MBSS) [5, 6] alongside flexible endoscopic evaluation of swallowing (FEES), which allows for continuous visualization of pharyngeal swallowing events, aspiration, and residue. Both of these visualization techniques are well-established and are essential for dysphagia diagnostics, but access is resource-limited due to expensive equipment in fixed locations (e.g., radiology, hospital, endoscopy), availability of trained personnel (e.g., speech-language pathology) and operators (e.g., radiologists for MBSS).

Ultrasound imaging offers advantages of portability, wide availability, low cost, and noninvasiveness, and has been evaluated as a tool for bedside care for decades [7–12]. Prior research has demonstrated ability of ultrasound to track hyoid and lingual movements during swallowing [13–19]. Most notably, when an ultrasound transducer is placed under the chin (submentally), B-mode ultrasound can visualize two structures key to oropharyngeal swallowing in real time: the tongue surface along its length from its blade to root and the hyoid bone, pulled into motion by muscular attachments to the tongue, epiglottis and larynx. The tongue surface is visible as a contour of bright reflections from the tongue-air interface, while the hyoid bone is visible due to its specular reflection and distal hypoechoic shadow.

However, adoption of ultrasound has been stymied by a lack of research establishing its clinical utility for dysphagia. As a recent critical review indicated, “there is burgeoning evidence to support [ultrasound] as an adjunct to. clinical assessment of swallowing and laryngeal function. However, … further research is required to establish reliability in [ultrasound] assessment as well as clear clinician-driven protocols and training” [14]. Key questions include whether ultrasound “provides similar information to... [MBSS] and FEES for swallowing assessment,” as well as resolution of issues with measurement reliability, probe positioning, and image acquisition [14].

Because of its clear and consistent visibility on both B-mode ultrasound and videofluoroscopy, the hyoid bone is an ideal starting point to assess congruence between ultrasound and videofluoroscopic measurements. Several previous studies have assessed the reliability of ultrasound for measuring hyoid bone displacements compared to videofluoroscopy [20–23], but several factors have limited their usefulness. Because swallowing is highly variable from bolus-to-bolus within and across individuals [24], natural variability between swallows can be a confounding factor. Some studies have compared non-simultaneous ultrasound and videofluoroscopic imaging (i.e., swallows of equivalent bolus at different times) [20, 21], and thus incorporated this natural variability. Two recent studies using simultaneous imaging reported challenges with image quality due to transducer choice and concerns with transducer stability [22, 23]. All these studies concentrated on scalar variables of overall hyoid displacement [20–23] and hyoid burst velocity [20]. Such measurements, taken at two distinct time points, do not fully characterize the complexity of hyoid motion, and are insufficient to assess the reliability of ultrasound throughout the entire swallow. These studies also used freehand ultrasound without correction for unstable transducer placement, caused by both clinician operator arm positioning [23] and spontaneous patient movements such as head motion, laryngeal excursion, and mastication, resulting in additional unknown errors in ultrasound measurements. Rigorous validation of ultrasound measurements requires not only direct comparison with simultaneous videofluoroscopic measurements, but also consideration of time-dependent hyoid motion, characterization of measurement errors due to transducer motion, and an approach to compensation for these motion-induced errors.

In this study, we address these barriers by analyzing simultaneous ultrasound and videofluoroscopy imaging from the same individuals performing multiple swallows with different standardized bolus types. To test our overall hypothesis that motion-corrected ultrasound measurements of hyoid movement can be equivalent to measurements from simultaneously recorded videofluoroscopy, we sought to quantitatively validate which measurements were equivalent. Time-dependent ultrasound array motion is measured from videofluoroscopic imaging, enabling both correction of measured movements and characterization of motion effects on measurement reliability. Ultrasound measurements are assessed based on agreement of time-dependent, two-dimensional hyoid motion, hyoid peak velocity magnitude, and overall hyoid excursion. This validation of ultrasound measurements is an important step toward evaluating the potential clinical utility of ultrasound as a tool for bedside characterization of oropharyngeal swallowing.

## Materials and Methods

### Patients and Participant Selection

All study procedures were approved by the Institutional Review Board of the University of Cincinnati (protocol #2021-0426). Adult patients (18 years or older) scheduled for a standard-of-care Modified Barium Swallow Study (MBSS) were eligible to enroll, with informed consent confirmed by written signature. Individuals were excluded if they were under the age of 18, pregnant, or unable to answer consent form comprehension questions. Acquisition of image data during standard-of-care MBSS helped to ensure that ultrasound measurements could be validated vs. MBSS measurements across a range of swallowing behaviors, including both normal and abnormal swallowing. Ultrasound and videofluoroscopic data acquisition then took place during the standard-of-care MBSS procedure, described in detail below.

### Acquisition and Analysis of Simultaneous Ultrasound and MBSS

Modified barium swallow study (MBSS) procedures were performed in a standard fluoroscopy suite, using a Shimadzu FLUOROSpeed X1 Patient-side R/F system (Shimadzu Scientific Instruments, Kyoto, Japan). Continuous videofluoroscopy imaging was recorded at 30 frames per second on a TIMS 2000 SP DICOM System (Foresight Imaging LLC, Chelmsford, MA) using TIMS MVP Software (versions 4.2 and 4.5) with simultaneous audio recording. Following standard of care, all simultaneous MBSS and ultrasound procedures followed the ALARA (as low as reasonably achievable) principle. The SLP conducting the ultrasound imaging wore appropriate leaded garments for protection from scattered radiation, following our institutions’ standard-of-care protocols for MBSS, which also specify quarterly checks of radiation dosimetry badges.

Bolus administration during MBSS was guided by a standard clinical protocol (Modified Barium Swallow Impairment Profile, MBSImP) [25] for bolus viscosity and delivery, with some boluses repeated to attain adequate image quality for ultrasound or MBSS. The bolus protocol, including safety and needs of the patient as well as data acquisition, was managed by the clinical SLP. Administration of bolus included the standard barium contrast materials (Varibar^®^ E-Z-EM, Inc. 40% w/v ratio, thin (International Dysphagia Diet Standardization Initiative, IDDSI 0), mildly thick (IDDSI 2), moderately thick (IDDSI 3), and Varibar pudding (IDDSI 4+).

Ultrasound image sequences, with synchronized audio recordings, were acquired during the MBSS by an SLP trained by the first and senior authors in ultrasound imaging of swallowing. For each bolus, a handheld ultrasound transducer was positioned submentally in the sagittal plane, just before the patient was prompted to swallow. The SLP sought to position the transducer to consistently view the hyoid bone and to limit transducer motion during swallowing. Participant comfort was monitored throughout each visit.

Ultrasound imaging employed a Telemed MicroUS Ext-1 H Compact External Ultrasound System (Telemed Ultrasound Medical Systems, Vilnius Lithuania) controlled by Echo Wave II software running on a Microsoft Windows-based laptop computer. Three curved linear array transducers were used, including the Telemed MicrUS C5-2R60S-3, MC8-4R20S-3, and MC4-2R20S-3, all using an imaging frequency of 4 MHz. Image quality was found to be comparable between these three arrays. The chosen transducer and scanner settings, with imaging depths ranging from 90 to 150 mm and ultrasound frame rates of 30–41 frames per second, were chosen to optimize imaging for each participant based on the clinician’s judgment. Ultrasound video and audio data were recorded from the laptop using OBS Studio screen recording software (version 29, Open Broadcaster Software Project) at 30–60 frames per second (fps).

Swallow function was assessed by two trained, licensed SLPs who assessed videofluoroscopic image sequences using the Penetration-Aspiration Scale (PAS) [26] using an exact-agreement, consensus approach. All MBS sequences were rated in duplicate by two independent raters, and exact agreement on each rating was reached on all non-congruent scores. Participants were considered to aspirate if their maximum (i.e., worst) PAS score was greater than 5, consistent with previous findings that normal healthy adults occasionally have patterns of penetration, but not aspiration [27].

Ultrasound and MBSS video clips were synchronized by the first author, combining both into a side-by-side video for each study. For each study, ultrasound and MBSS recordings were opened as clips in a video editing software program (DaVinci Resolve 18, Blackmagic Design). If necessary, ultrasound video clips were downsampled to 30 fps using nearest-neighbor interpolation to match the videofluoroscopy frame rate. Video clips were flipped so that the anterior anatomic direction faced right to match fluoroscopy images. Audio and video landmarks detectable in both ultrasound and fluoroscopy recordings were then identified as reference points for synchronization. Video landmarks included placement and withdrawal of the ultrasound transducer, initiation of hyoid movement, and attainment of the furthest anterior hyoid position. Audio landmarks, identified aurally and timed based on plotted waveforms, included verbal prompting of swallows by the SLP and electronic beeps produced by the fluoroscopy system. The shorter video was then advanced or delayed in time, such that each landmark shared the same time stamp, with an approximate precision of 1 ultrasound frame duration (33 ms). The resulting combined synchronized video was then saved with ultrasound and MBSS appearing side-by-side, with each taking up approximately half the video frame, and separated into clips for further analysis. Each clip included only one swallow, with the swallow’s beginning and end defined as the first and last frames where the hyoid was both visible and stable on both ultrasound and MBSS.

### Final Inclusion Criteria

To simplify video synchronization and comparisons between different subjects, only swallows employing standardized bolus volumes of 5 ml (thin, mildly thick [nectar], moderately thick [honey], and pudding textures) were analyzed. Analysis and measurements were only completed for video sequences that included sufficient audio and visual landmarks for adequate synchronization, for which the hyoid bone was depicted in all ultrasound and fluoroscopy frames, and for which the C3 vertebra and ultrasound array were visible in all fluoroscopy frames.

### Measurements

For measurements of swallowing movements, motion of anatomic landmarks on ultrasound and MBSS videos was semi-automatically tracked by the first author using CASM (Computer Analysis of Swallowing Mechanics, version 5.1 beta) [28] running within the MATLAB environment (version 2023a, The MathWorks). The tracking method employed placement of a reference marker on the boundary of an anatomic landmark in the initial video frame, which is then tracked in subsequent frames using a Harris corner detector [29]. If semi-automatic tracking visibly failed to localize a landmark for any video frames, reference points were placed manually on the frames in question. To enable direct comparison between ultrasound and MBSS measurements, an equal number of frames was tracked for both modalities in a given swallow.

**Fig. 1:**
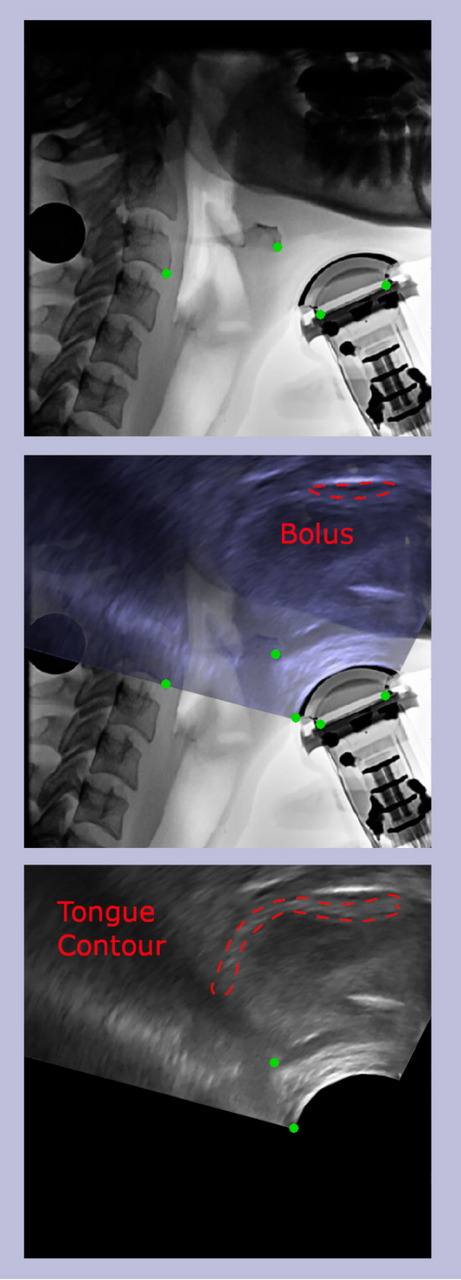
Representative synchronous ultrasound and videofluoroscopic (MBSS) imaging. Top: MBSS image frame. Bottom: Labeled ultrasound B-mode frame after registration to MBSS. Middle: Co-registered, blue-tinted B-mode ultrasound frame overlaid onto MBSS frame. Green dots in each panel correspond with tracked landmarks

For both ultrasound and MBSS, the hyoid bone was tracked at the most inferior point of its medial ridge. Additional features tracked on the fluoroscopy video included the C3 vertebra as well as two internal edges of the ultrasound transducer. To correct for head motion in MBSS videos, the time-dependent tracked position of the C3 vertebra was subtracted from the time-dependent hyoid positions measured from the videofluoroscopic images. Figure 1 shows tracked structures in a B-mode ultrasound image, tinted blue and overlaid onto a synchronous fluoroscopy image.

To measure the time-dependent motion of the ultrasound transducer, two interior reference points, marked in Fig. 1, were tracked on the MBSS video frames. From these reference points, the time-dependent transducer angle and position of a defined origin point (0,0) were determined for the entire videofluoroscopic image sequence. To characterize motion of the handheld transducer and resulting errors in measurements of tissue movements, means and standard deviations of the transducer angle, horizontal displacement, and vertical displacement were computed for each swallow, as well as root-mean-square values of these standard deviations across all swallows to characterize overall variability of transducer motion.

Outputs from CASM comprised lists of frame-by-frame landmark positions, measured in pixels relative to the bottom-left corner of the video frame. Positions were converted into centimeter units using the included scale bar from the ultrasound video and a reference object (penny) placed on the participant’s neck in the fluoroscopy video. Reference distances for scale calibration were measured in ImageJ (Version 1.54, US National Institutes of Health) [30].

**Fig. 2:**
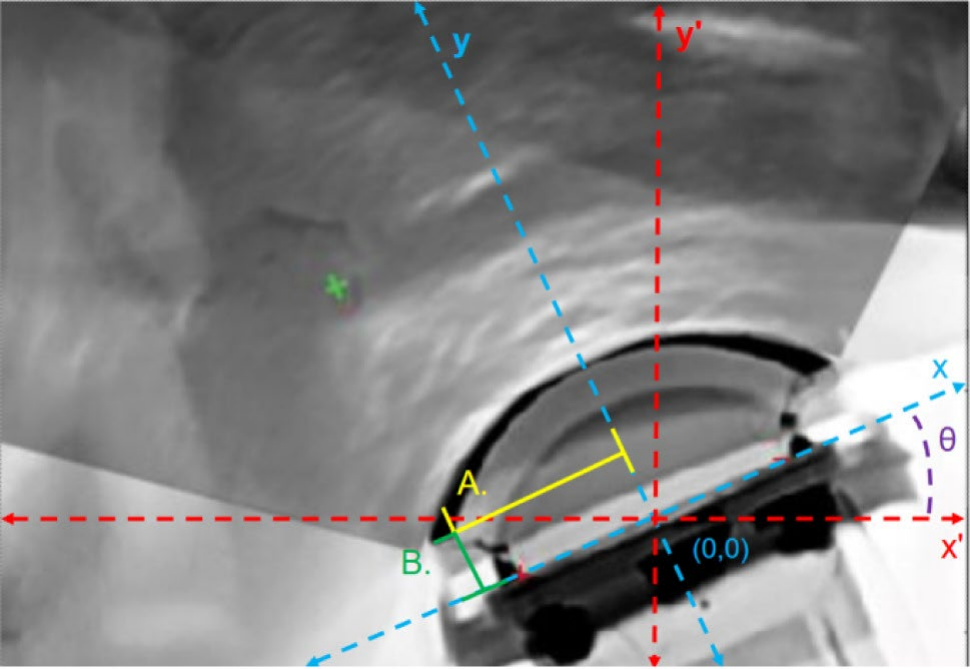
Axes and measurements for registration of ultrasound images to videofluoroscopic imaging. Axes (*x*,*y*) and (*x*’,*y*’) respectively correspond to Cartesian coordinate systems for B-mode ultrasound and videofluoroscopy image sequences, with the common origin (0,0) defined as the position of a tracked reference point within the transducer at the initial frame. The rotational angle between axes of these Cartesian coordinate systems is θ. (**A**) Line segment used to measure horizontal (*x*) distance from tracked corner of B-mode images to the origin. (**B**) Corresponding line segment used to measure vertical (*y*) distance from tracked corner of B-mode images to the origin

**Fig. 3:**
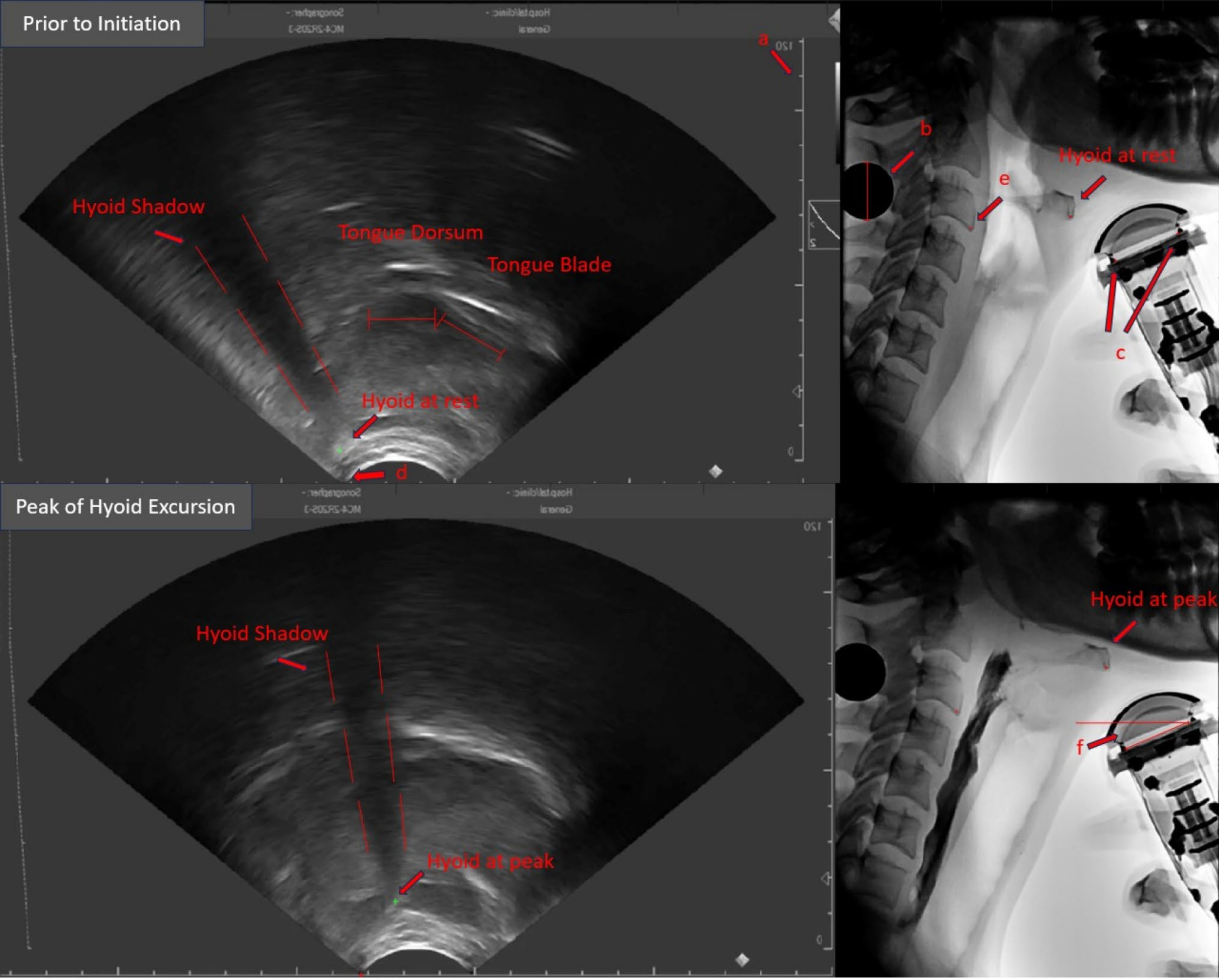
Still frames from ultrasound (left) and MBSS (right), with labeled features and correction components. (**A**) Ultrasound length calibration scale. (**B**) MBSS length calibration scale. (**C**) Internally tracked transducer positions used for calculation of transducer movement. (**D**) Coordinate reference for correction of ultrasound displacement data. (**E**) Tracked C2 vertebra used for head movement correction. (**F**) Angle used to correct ultrasound measurements to MBSS-derived room coordinates

To test the feasibility and performance of methods correcting for transducer motion, two correction approaches were applied to ultrasound measurements. The first, called static rotation, rotated the ultrasound video so that its initial frame was aligned with the videofluoroscopy (room) coordinates, seen as the (*x*’,*y*’) axes in Fig. 2. The second method, called dynamic translation/rotation, additionally corrected for the time-dependent translation and rotation of the ultrasound array as measured from the videofluoroscopic image sequence. Both these correction methods could also be implemented without use of videofluoroscopy, such as by measuring transducer position and angle either with a fixed optical camera or with motion sensors integrated into the transducer. Figure 3 shows two representative tracked ultrasound frames with labeling of features including the hyoid bone, transducer reference points, C3vertebra, measured array angle, and calibration length scales.

For ultrasound measurements, the relative hyoid bone displacement was determined from CASM tracking data using the transformation

1 $$\:\left[\begin{array}{c}{\Delta}{H}_{X}(t)\\\:{\Delta}{H}_{Y}(t)\end{array}\right]=\left[\begin{array}{cc}\mathrm{cos}\:\theta(0)&\:-\mathrm{sin}\:\theta(0)\\\:\mathrm{sin}\:\theta(0)&\:\:\:\:\:\mathrm{cos}\:\theta(0)\end{array}\right]\left(\left[\begin{array}{c}{X}_{H}(t)\\\:{Y}_{H}(t)\end{array}\right]-\left[\begin{array}{c}{X}_{H}(0)\\\:{Y}_{H}(0)\end{array}\right]\right)$$

for correction using static rotation, or alternatively, the transformations

2 $$\begin{aligned}\left[\begin{array}{c}{H}{X}(t)\\\:{H}{Y}(t)\end{array}\right]=& \left[\begin{array}{cc}\mathrm{cos}\:\theta(t)&\:-\mathrm{sin}\:\theta(t)\\\:\mathrm{sin}\:\theta(t)&\mathrm{cos}\:\theta(t)\end{array}\right]  \left[\begin{array}{c}{X}{H}(t)\\\:{Y}{H}(t)\end{array}\right]\\ &\!\!\!\!\!\!\!\!\!\!\! \quad+\left[\begin{array}{c}{T}{X}(t)\\\:{T}{Y}(t)\end{array}\right]-\left[\begin{array}{c}{C}{X}(t)\\\:{C}{Y}(t)\end{array}\right], \\ \:\left[\begin{array}{c}{\Delta}{H}{X}(t)\\\:{\Delta}{H}{Y}(t)\end{array}\right]=&\left[\begin{array}{c}{H}{X}(t)\\\:{H}{Y}(t)\end{array}\right]-\left[\begin{array}{c}{H}{X}(0)\\\:{H}{Y}(0)\end{array}\right]\end{aligned}$$

for correction using dynamic translation/rotation. In these equations, $\:\theta(t)$ is the time-dependent transducer angle measured from videofluoroscopy, seen in Fig. 2, $\left[{X}_{H}(t),{Y}_{H}(t)\right]$ is the hyoid position in an ultrasound image frame relative to the assigned origin point (corresponding to the tracked transducer position in the videofluoroscopic image sequence), $\left[{T}_{X}(t),{T}_{Y}(t)\right]$ is the transducer position measured from videofluoroscopy, $\left[{C}_{X}(t),{C}_{Y}(t)\right]$ is the C3 vertebra position from videofluoroscopy, $\left[{H}_{X}(t),{H}_{Y}(t)\right]$ is the hyoid position measured by ultrasound after transformation to room (videofluoroscopy) coordinates and correction for transducer and C3 vertebra displacement, and $\left[{\Delta}{H}_{X}(t),{{\Delta}H}_{Y}(t)\right]$ is the corrected relative hyoid displacement measured from ultrasound.

Hyoid kinematics, tracked using CASM as described above, were analyzed using MATLAB. The time-dependent hyoid displacement was first smoothed using a three-point moving mean to reduce effects of repeated or skipped frames. Time-dependent hyoid velocity was calculated as a time-dependent vector with components in the posterior-anterior and inferior-superior directions, obtained using second-order accurate centered differences applied to the respective displacement time series. Velocity magnitude was computed as the Pythagorean sum of the two velocity components, and its temporal maximum was recorded for each swallow. Hyoid excursion distance was defined as the Euclidean distance between the furthest anterior and posterior hyoid positions during a swallow.

### Statistical Analysis

Statistical analysis was done using MATLAB and R (4.2.0, R Core Team 2023) [31]. To validate the accuracy of ultrasound measurements vs. gold-standard MBSS, root-mean-square (RMS) error of the time-dependent hyoid displacement was calculated as the RMS Euclidean distance between displacement trajectories measured from ultrasound and fluoroscopy. RMS error was calculated for each swallow, as well as over the set of all analyzed swallows. Per-swallow RMS error and its variance was compared between the two ultrasound correction methods using a paired *t* test and a two-sample *F* test respectively.

As a second measure of validity, intraclass correlation coefficients (ICC) were computed between ultrasound- and MBSS-measured hyoid displacement trajectories using a two-way, mixed effects model for absolute agreement [32]. ICCs were similarly computed for the maximum velocity magnitude and the overall hyoid excursion for each swallow. Values of the ICC were interpreted with ICC < 0.5, 0.5 < ICC < 0.75, 0.75 < ICC < 0.9, and ICC > 0.9 taken to indicate poor, moderate, good, and excellent reliability respectively [32].

Comparisons between measured hyoid motion statistics were made for the overall hyoid excursion and maximum hyoid velocity magnitude. The mean and standard deviation of these values were compared separately using paired *t* tests for each considered bolus for MBSS, ultrasound corrected using static rotation, and ultrasound using dynamic translation/rotation. These summary statistics computed from ultrasound and MBSS measurements were also compared between grouped individuals without aspiration (maximum PAS scores 5 or less) and with aspiration (maximum PAS scores 6 or greater) using unpaired *t* tests.

**Table 1:** Participants’ sex, age, primary diagnosis related to ordered MBSS, race, ethnicity, and clinically evaluated maximum (worst) penetration-aspiration scale (PAS) score

ID	Sex	Age (y)	Diagnosis	Race	Ethnicity	Max PAS
1	F	28	Globus	White	Not Hispanic	2
2	F	42	GERD, Pneumonia	White	Not Hispanic	2
3	M	53	PD, GERD	White	Not Hispanic	8
4	M	68	TBI, COPD	White	Not Hispanic	2
5	F	50	GERD/LPR, Stroke, Chronic bronchitis	AA	Not Hispanic	1
6*	M	78	Acquired neuromuscular disease, Heart problem/disorder, Spinal cord injury	White	Not Hispanic	6
7*	M	47	Stroke	White	Not Hispanic	7
8	M	54	Globus	African- American	Undisclosed	1
9	F	56	GERD, TBI	Undisclosed	Undisclosed	3
10	F	86	Stroke, Pain in throat	AA	Not Hispanic	5
11	M	69	GERD/LPR	White	Not Hispanic	2
12*	M	71	Pneumonia, Heart problems/disorders	White	Not Hispanic	2
13	M	63	GERD/LPR, Heart problems/disorders	AA	Not Hispanic	8
14	F	33	Globus	White	Not Hispanic	1
15	M	51	Pneumonia, Thyroid Cancer	AA	Not Hispanic	2
16	F	49	Frequent Heartburn	AA	Not Hispanic	2
17	M	68	GERD	White	Not Hispanic	2
18	M	47	Stroke	White	Not Hispanic	8
19	F	37	GERD	White	Not Hispanic	2
20*	F	53	GERD	White	Not Hispanic	1
21	M	74	Frequent throat clearing	White	Not Hispanic	8
22	F	71	Globus	White	Not Hispanic	1
23	M	64	GERD	White	Not Hispanic	1

*Participant data not included in final analysis

**AA: AA;** GERD: gastroesophageal reflux disease, TBI: traumatic brain injury, PD: Parkinson’s disease. LPR: laryngopharyngeal reflux

To characterize motion of the ultrasound array during freehand imaging of swallowing, similar statistics were computed. The per-swallow mean and standard deviation of the measured transducer angle, horizontal displacement, and vertical displacement were first computed separately for each swallow. Overall means and standard deviations were then computed as the average of the per-swallow means and the RMS value of the per-swallow standard deviations, respectively. The resulting statistics indicate both the typical overall motion of the handheld ultrasound array during a swallow and the variation of this typical motion among multiple swallows.

## Results

### Participants

Out of 29 patients eligible for enrollment in this study, 25 individuals consented to participation. Two of these individuals were withdrawn after their initial participation. One voluntarily withdrew, while the other was withdrawn after data collection because their MBSS video data was not correctly recorded.

Participant characteristics are summarized in Table 1. A total of 23 participants completed the study, yielding a total of 92 possible swallows with 5 ml boluses. Ultrasound and MBSS data from 56 swallows, performed by 19 distinct participants, met the criteria for analysis. Swallows were excluded for reasons including incomplete recording (1 swallow), inability to synchronize due to insufficient audio-visual landmarks (3 swallows), and the hyoid bone not remaining visible for tracking in all ultrasound image frames due to loss of array-skin contact, movement outside the ultrasound image frame, or poor image resolution (28 swallows). Thus, ultrasound imaging of the hyoid was sufficient for comparison of ultrasound and videofluoroscopic measurements in two thirds of all synchronized video sequences. After analysis of these 56 swallows, 1 swallow was additionally excluded from the dynamic translation/rotation method of ultrasound measurement correction, due to tracked transducer reference points falling outside one or more MBSS image frames.

**Fig. 4:**
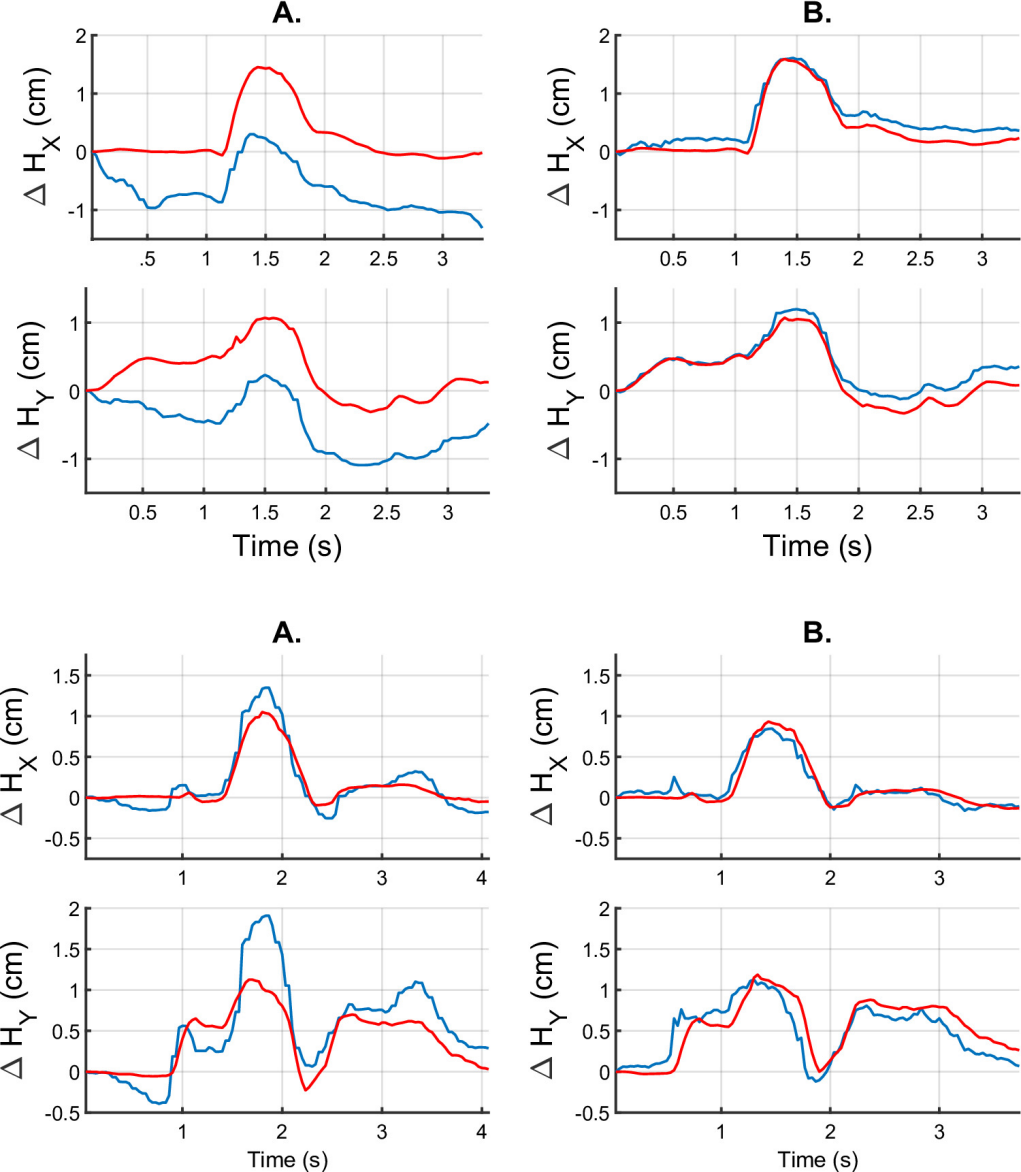
Time-dependent anterior and superior hyoid displacement for two representative 5 ml thin swallows, with ultrasound measurements in blue and MBSS measurements in red. Top: swallow 1, participant 1. Bottom: swallow 2, participant 19. (**A**) MBSS vs. ultrasound with static angle correction. (**B**) MBSS vs. ultrasound with dynamic translation/rotation

Using the MBSImP protocol, the 19 participants whose data were included for analysis were assigned a clinical grading of 1–8 on the PAS scale, with 4 participants scoring as aspirating (worst PAS score 6–8; material entering airway) and the remaining 15 participants scoring within the range of worst PAS scores 1–5 (no aspiration of material past the vocal folds).

**Fig. 5:**
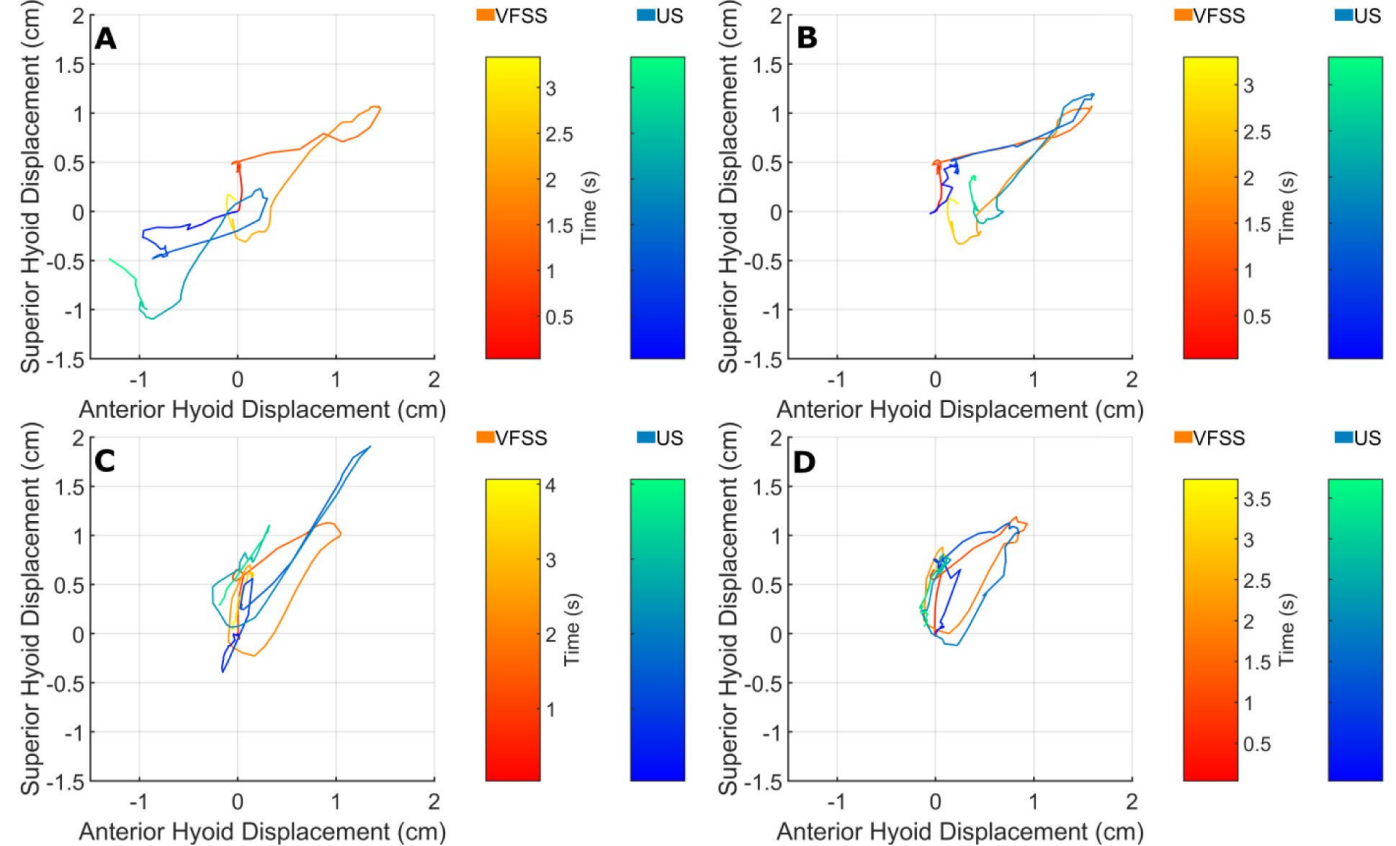
Parametric plots of time-dependent hyoid trajectories measured by ultrasound and MBSS. Vertical (inferior-superior) displacement is plotted vs. horizontal (posterior-anterior) displacement with elapsed time indicated by line color. (**A**) Swallow 1, participant 1; static angle correction. (**B**) Swallow 1, participant 1; dynamic translation/rotation. (**C**) Swallow 2, participant 19; static angle correction. (**D**) Swallow 2, participant 19; dynamic translation/rotation

### Validation of Ultrasound vs. MBSS Measurements

Time-dependent hyoid displacements, measured by MBSS and by ultrasound with the two position-correction methods employed, are seen in Figs. 4 and 5 for two representative thin swallows (5 ml thin barium bolus). Time-dependent horizontal (posterior-anterior) positions are plotted in Fig. 4, while hyoid trajectories are shown as parametric plots in Fig. 5. Generally, ultrasound measurements corrected by dynamic translation/rotation corresponded closely with MBSS measurements, while ultrasound measurements corrected by only static rotation showed errors that accumulated with time during some swallows.

**Fig. 6:**
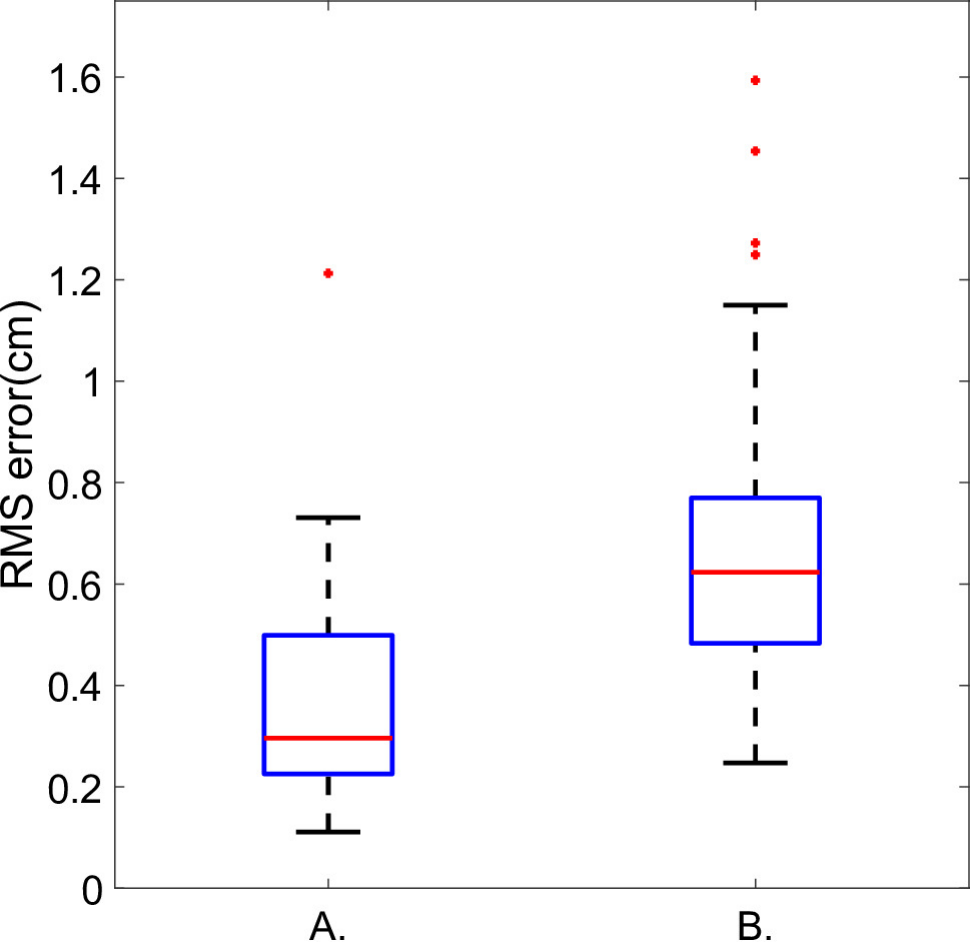
Box plots of RMS error between ultrasound and MBSS measurements of time-dependent hyoid position, with red lines indicating the median, dotted whiskers indicating the interquartile range, and individual red dots representing outliers. (**A**) Ultrasound measurements corrected using dynamic translation/rotation. (**B**) Ultrasound measurements corrected using static angle correction

RMS error of time-dependent hyoid displacement for ultrasound- vs. MBSS-based measurements for each analyzed swallow are shown as box plots in Fig. 6. For all 56 swallows, correction by static rotation resulted in RMS displacement error of 0.771 cm between ultrasound and MBSS measurements. For the 55 of these swallows where ultrasound measurements were also corrected using dynamic translation/rotation, RMS error reduced to 0.3795 cm, a significant difference (*p* = 5.52 × 10^−10^, two-sided). Variance of the RMS error among all swallows was also significantly smaller for dynamic translation/rotation compared to static angle correction (*p* = 0.0062, two-sided).

Intraclass correlation coefficients (two-way, mixed model for absolute agreement) for hyoid positions were 0.503 (95% confidence interval [CI] 0.485–0.520) when comparing ultrasound measurements corrected by static rotation vs. MBSS measurements, indicating poor to moderate agreement. In contrast, ICC when for ultrasound corrected by dynamic translation/rotation vs. MBSS was 0.891 (95% CI 0.886–0.895), indicating excellent agreement. Both ICC values were significantly higher than chance expectations (*p* < 10^−16^).

**Fig. 7:**
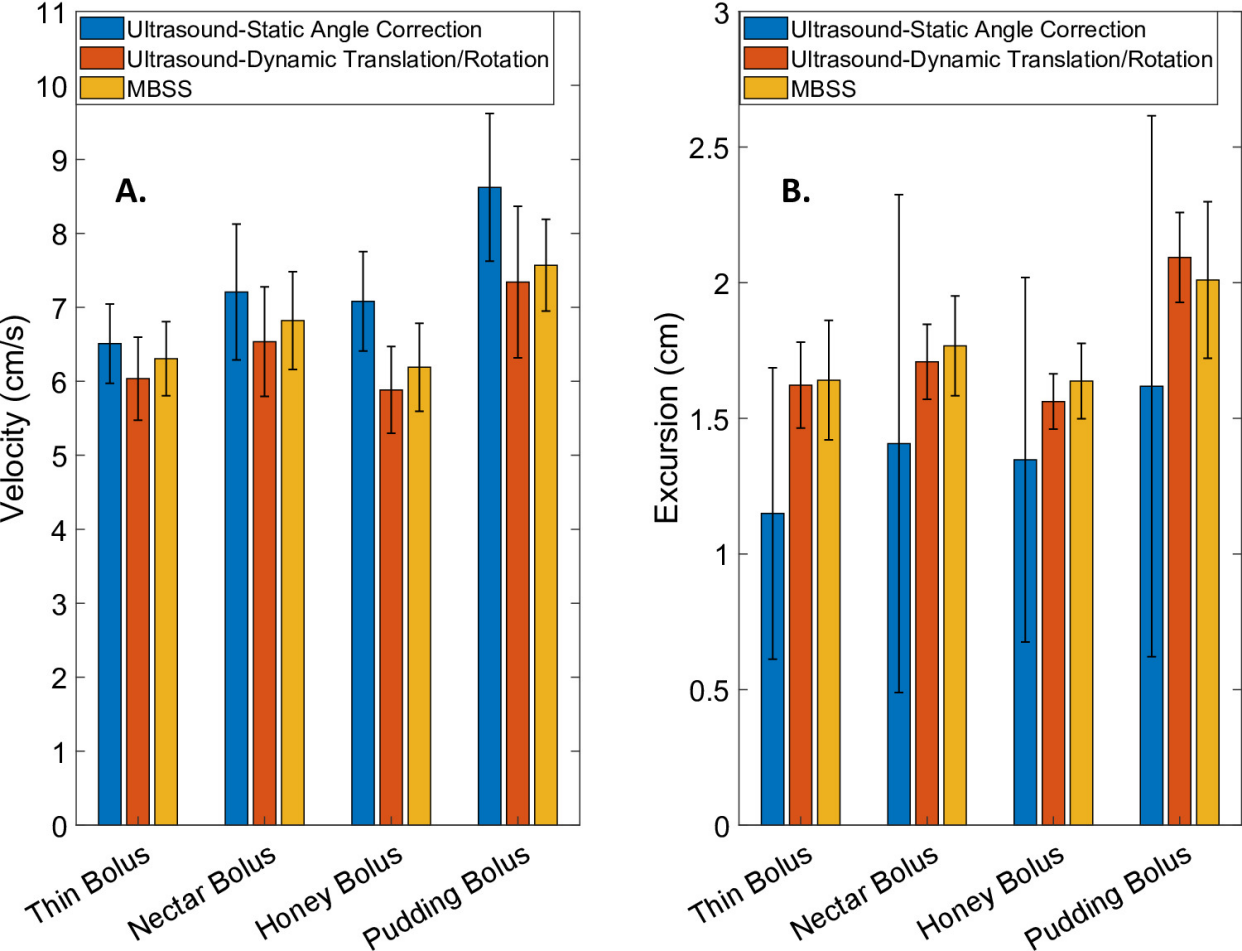
Means and standard errors of hyoid motion measures for MBSS and ultrasound correction by both static rotation and dynamic translation/rotation (*N* = 55). (**A**) Maximum hyoid velocity magnitude per swallow. (**B**) Overall hyoid excursion

**Table 2:** Mean maximum hyoid velocity magnitude and standard error for each bolus thickness, as measured by ultrasound (US) with two motion correction methods and by MBSS

Hyoid Velocity Magnitude (cm/s)				
**Modality**	Thin (*N*=16)	Nectar (*N*=16)	Honey (*N*=13)	Pudding (*N*=10)
Static angle corrected US	$6.51\pm\:0.54\:$	$7.21\:\pm\:0.92$	$7.08\:\pm\:0.67$	$8.62\:\pm\:1.00$
Dynamic translation/rotationcorrected US	$6.03\:\pm\:0.56$	$6.53\:\pm\:0.74$	$5.88\:\pm\:0.59$	$7.34\pm\:1.03$
US	$6.30\:\pm\:0.50$	$6.82\:\pm\:0.66$	$6.18\:\pm\:0.60$	$7.57\:\pm\:0.62$

Comparison swallow summary statistics, illustrated in Fig. 7, were not significantly different between MBSS and ultrasound when correcting using dynamic translation and rotation, but were significantly different for static angle correction. Statistics comparing maximum hyoid velocity magnitude as measured by ultrasonography and MBSS are displayed in Table 2, while statistics comparing overall hyoid excursion are shown in Table 3. Overall hyoid excursion was significantly different between MBSS and ultrasound with static angle correction (paired *t* test, *N* = 55, *p* = 2.14 × 10^− 4^), but not with dynamic translation/rotation (*N* = 55, *p* = 0.764). Similar trends were observed for measurements of the maximum hyoid velocity magnitude (*p* = 0.0173 for static angle correction, *p* = 0.125 for dynamic translation/rotation). ICC values for the overall hyoid excursion vs. MBSS were 0.577 (95% CI 0.369–0.730, *p* = 1.95 × 10^− 6^) for ultrasound corrected by dynamic translation/rotation and 0.442 (95% CI 0.166–0.646, *p* = 0.00119) for ultrasound corrected by static rotation. Corresponding ICCs for the maximum hyoid velocity magnitude were 0.852 (95% CI 0.759–0.911, *p* < 10^− 10^) for dynamic translation/rotation and 0.762 (95% CI 0.613–0.856, *p* < 10^− 10^) for static angle correction.

Because this work aimed to assess agreement between MBSS and ultrasound tracking of the hyoid for a range of swallows, we analyzed data from participants undergoing standard-of-care MBSS. These included individuals with normal, mild, and severe swallowing impairment. Of the 19 participants with data included for analysis, 3 were found to show aspiration (maximum PAS scores greater than 5). Although this small number of participants with high PAS scores limited comparisons between individuals with varying degrees of dysphagia, some trends in summary statistics of hyoid motion were observed. Overall hyoid excursion was significantly higher in swallows by individuals with aspiration (*N* = 8) compared to swallows by individuals without aspiration (*N* = 47) for measurements using MBSS (*p* = 0.0042) or using ultrasound with motion correction by dynamic translation and rotation (*p* = 0.0239). Maximum hyoid velocity magnitude was also significantly higher in swallows by individuals with aspiration for measurements using MBSS (*p* = 0.0186) or using ultrasound with motion correction by dynamic translation and rotation (*p* = 0.0342).

**Table 3:** Mean hyoid excursion and standard error for each bolus thickness, as measured by ultrasound (US) with two motion correction methods and by MBSS

Hyoid Excursion (cm)				
**Modality**	Thin (*N* = 16)	Nectar (*N* = 16)	Honey (*N* = 13)	Pudding (*N* = 10)
Static angle corrected US	$1.15\:\pm\:0.54$	$1.41\:\pm\:0.92$	$1.34\:\pm\:0.67$	$1.62\:\pm\:1.00$
Dynamic translation/rotation corrected US	$1.62\:\pm\:0.16$	$1.71\:\pm\:0.14$	$1.56\:\pm\:0.10$	$2.09\:\pm\:0.17$
MBSS	$1.64\:\pm\:0.22$	$1.77\:\pm\:0.18$	$1.64\:\pm\:0.14$	$2.01\:\pm\:0.29$

**Fig. 8:**
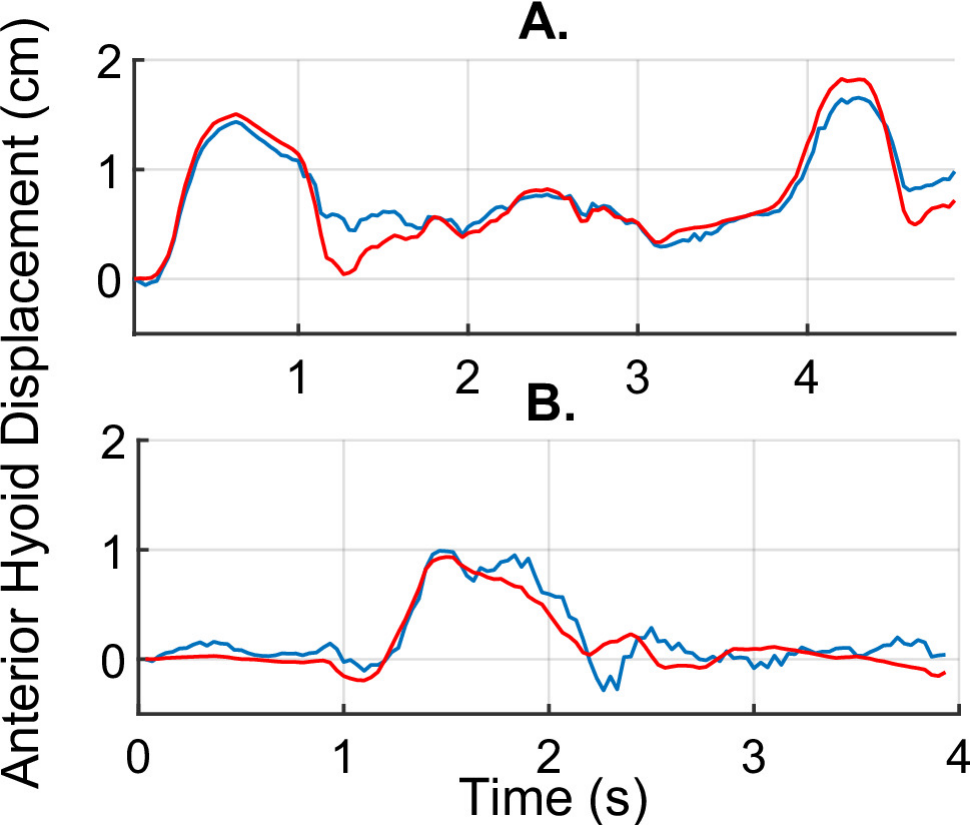
Time-dependent anterior hyoid displacement for representative 5 ml thin swallows, with ultrasound measurements in blue and MBSS measurements in red. (**A**) Representative swallow by a participant with severe dysphagia, showing extended hyoid excursions (participant 18, PAS 8). (**B**) Representative swallow by a participant who did not aspirate during their study (participant 1, PAS 2)

Qualitative differences between individuals with varying degrees of dysphagia were also observed. For example, in Fig. 8, comparison of time-dependent anterior hyoid displacement for 5 mL honey swallows by a participant with substantial aspiration (worst PAS 8) and a typical participant without observed aspiration (worst PAS 2), shows repeated hyoid excursions for the individual with PAS 8 but a single, well-defined excursion for the individual with PAS 2. Similar patterns of hyoid displacement were seen in multiple participants, consistent with the observation of significantly higher overall hyoid excursion in individuals with aspiration.

### Quantification of Transducer Motion

**Fig. 9:**
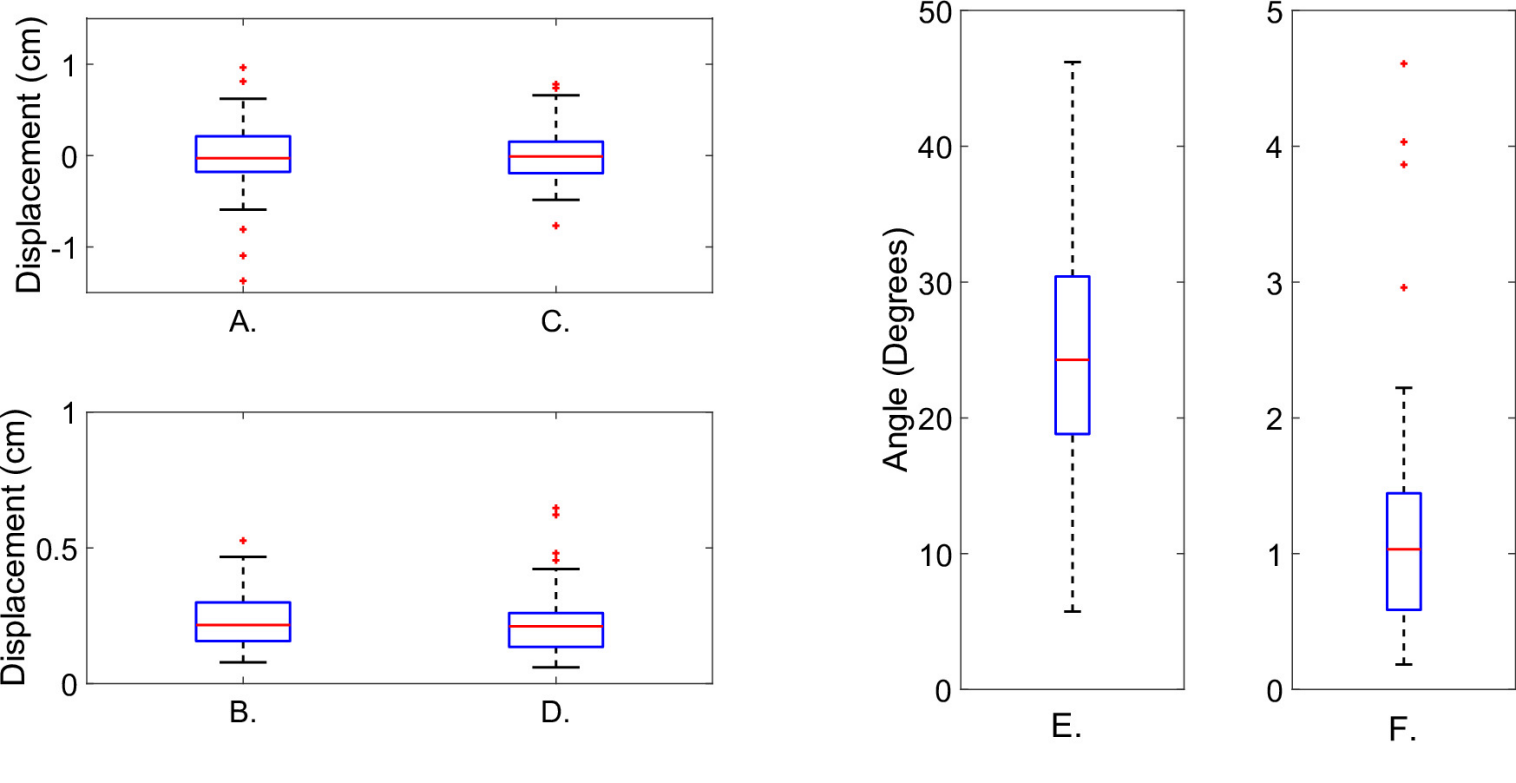
Box plots of summary statistics for ultrasound transducer motion measured by MBSS in *N*=55 swallows. Left: (**A**) Mean horizontal transducer displacement. (**B**) Mean vertical transducer displacement. (**C**) Standard deviation of horizontal transducer displacement. (**D**) Standard deviation of vertical transducer displacement. Right: (**E**) Mean transducer angle during swallow. (**F**) Standard deviation of transducer angle during swallow

The time-dependent angle, horizontal position, and vertical position of the handheld ultrasound array, measured by MBSS, are summarized in Fig. 9 by box plots of the per-swallow mean and standard deviation for each quantity. The overall average transducer angle was 24.48°, with an overall standard deviation of ± 1.57°. To interpret the effect of this rotation on the error of measured hyoid displacements, the original and rotated hyoid positions are noted to fall on a circle centered at the origin of rotation, so that the distance between these positions is the length of a chord spanning the transducer’s rotation angle. Thus, the RMS error in measured hyoid position resulting from transducer rotation is approximately

3 $$\:\mathrm{RMSE}=\:\left|2\,r\,\mathrm{s}\mathrm{i}\mathrm{n}\left(\theta/2\right)\right|,$$

where $\:\theta\:$ is the rotation angle and *r* is the distance of a rotated point from the origin of rotation.

In measurements of time-dependent transducer translation, the overall average displacement was 0.0595 cm in the horizontal (posterior-anterior) and −0.0784 cm in the vertical (inferior-superior) direction. Overall standard deviations of the transducer displacement, computed as RMS values of the per-swallow standard deviation, were 0.273 cm in the horizontal direction and 0.245 cm in the vertical direction.

## Discussion

Ultrasound tracking of swallowing movements has previously shown promise in tracking features of the swallow, especially hyoid bone motion [15, 18, 19, 21], but previous studies have not rigorously validated time-dependent ultrasound measurements against the clinical standard of videofluoroscopy. This study has evaluated the accuracy of ultrasound hyoid motion measurements vs. measurements from simultaneous videofluoroscopic imaging, assessed correlation and error for the time-dependent hyoid position and multiple related variables, and assessed two approaches to position corrections based on transducer angle and position measurements. Ultrasound measurements corrected using dynamic translation/rotation showed high reliability and relatively small error for tracking of the time-dependent hyoid displacement (ICC 0.891, RMS error 0.3795 cm for ultrasound vs. MBSS). For comparison of maximum hyoid velocity magnitude between ultrasound and MBSS, the ICC of 0.852 observed here using correction by dynamic translation/rotation was within the range found for the hyoid burst velocities and displacements in previous studies [18–21]. Correction of ultrasound measurements using static angle correction was less successful (ICC 0.503 and RMS error 0.771 cm for time-dependent hyoid position; ICC 0.762 for maximum hyoid velocity magnitude), due to accumulation of position errors during some swallows, as seen in Fig. 4. Values for maximum hyoid excursion and maximum velocity magnitude generated using static angle correction were also significantly different when compared to MBSS, (*p* = 2.14 × 10^− 4^ and *p* = 0.0173, respectively), which was not found for values generated with dynamic translation/rotation (*p* = 0.764 and *p* = 0.125, respectively). The overall hypothesis of this study, that motion-corrected ultrasound measurements of hyoid movement can be equivalent to measurements from simultaneously recorded videofluoroscopy, can thus be considered confirmed for ultrasound with motion correction by dynamic translation/rotation, but not for static angle correction.

Observed hyoid bone motion, as illustrated by the trajectories plotted in Fig. 6, were consistent with previous literature, with the hyoid bone traveling from its posterior position, moving in a superior direction, rapidly moving in an anterior direction, then returning posterior and inferior toward its initial position [18, 24]. Values measured here for the maximum hyoid excursion, averaging 1.6–2.0 cm with standard deviations 0.11–0.29 cm (Table 2), were comparable to those found by Ueda et al. (2013) (mean $\:\pm\:$ standard deviation 2.03 $\:\pm\:$ 0.36 cm), Ma and Wrench (2022) (1.60 $\:\pm\:$ 0.40 cm) and (2025) (1.10 $\:\pm\:$ 0.43 cm and 1.01 $\:\pm\:$ 0.47 cm for 5 and 10 mL water bolus), and Kim et al. 2021 (2.11 $\:\pm\:$ 0.67 cm).

Hyoid velocities observed here, with maximum magnitudes averaging 6–9 cm/s, fell within the lower end of the range 5–32 cm/s reported in previous literature [18, 19, 33–36]. Values were computed here using second-order accurate centered differences after smoothing by a 3-point moving average, which effectively implemented a low-pass filter with a −6 dB cutoff frequency of 6 Hz. Notably, measured velocity values depend on the method of calculation, which has varied between studies, with some defining velocity based on the overall distance and time traveled by the hyoid in its initial anterior advance [34] and others estimating instantaneous velocity by finite-difference analysis of the time-dependent hyoid position, with varying levels of differencing order and smoothing [18, 33–35, 37]. For example, Ueda et al. (2013) reported lower values for maximum velocity magnitude (4.99 $\:\pm\:\:$0.9 cm/s), likely due to the fourth-order-accurate, 5-point backwards difference function employed. Ma and Wrench (2022) calculated comparable values for the maximum anterior hyoid velocity (5.12 $\:\pm\:$ 0.18 cm/s) using finite differences after low-pass filtering, comparable to the smoothing employed here. Other studies have found higher peak velocities, such as average peak velocities of 16.7 cm/s for thin boluses [34] and 25.2–32.2 cm/s for multiple boluses [36]. However, these larger values were obtained based on measured hyoid displacement between sequential videofluoroscopic frames, equivalent to first-order accurate finite differences without any smoothing.

Differences in hyoid kinematics between boluses could also be seen within this dataset, with an overall trend of increased velocity and excursion distance for thicker boluses, consistent with the trends reported by Nagy et al. (2015) [38]. Both peak velocity and excursion distance, as measured by MBSS and ultrasound corrected using dynamic translation/rotation, were greater for nectar compared to thin boluses, while mean hyoid velocity and excursion distances were substantially larger for pudding boluses compared to thin, mildly thick, and moderately thick boluses, as seen in Fig. 8. However, the same quantities measured for moderately thick boluses, not included by Nagy et al. [34] were slightly smaller than those for nectar boluses. None of these differences attained statistical significance for the relatively small group sizes studied here.

Occurrence of repeated hyoid excursions in swallowing by individuals with dysphagia, as illustrated in Fig. 8, has been described in prior literature as well, such as Ref [13] (Sonies et al. 1996), which described “abnormalities characterized by multiple swallows and hesitations.” However, the disparate etiologies associated with dysphagia have differing effects on hyoid motion. For example, myopathy can greatly reduce the excursion of the hyoid bone [39], opposite to the trend of greater excursion seen here for subjects with higher maximum PAS scores. In addition, the number of individuals with aspiration in the current study was a small fraction of the overall cohort (*N* = 3 out of 23). In general, findings comparing hyoid motion between individuals with normal and abnormal swallowing should be interpreted with caution and considered in the context of broader individual pathology.

Even so, the presence of repeated hyoid excursions suggests the potential quantitative measure of hyoid travel distance, which could be calculated by integrating the magnitude of hyoid displacement. Hyoid travel distance, quantifying the overall movement of the hyoid bone during a swallow, may increase with repeated hyoid excursions and could be indicative of greater effort or dysphagia. Further integral or derivative-based metrics may also be feasible to enhance the understanding of hyoid dynamics.

Quantitative measurements of transducer motion in this study illustrate sources of error that can affect ultrasound imaging from the submental array position. Although head stabilizers are widely used for submental ultrasound image measurements of speech production [40], such devices could impact the swallowing process by interfering with natural anatomic motion. The average transducer angle measured here was 24.48° from horizontal, estimated from Eq. (3) to contribute approximately 0.14 cm to the RMS error of measured hyoid position. The overall standard deviation of transducer displacement, measured here as 0.27 cm in the horizontal direction and 0.25 cm in the vertical direction, can be taken as an estimate of additional RMS error to hyoid displacement measurements. The Pythagorean sum of these errors yields an estimated 0.37 cm contribution of uncorrected transducer motion to the overall RMS error of hyoid position measurements. This error reduces the accuracy of ultrasound measurements and may also impact the identification of hyoid movement patterns and statistics such as velocity and hyoid excursion.

Notably, although correction for transducer motion was performed here using MBSS measurements of array position, similar correction could be performed without fluoroscopy. Transducer position could potentially be measured using an optical camera with tracking of visible markers or other landmarks on the array, or by the use of accelerometer and gyroscope sensors, either incorporated into the array [41] or appropriately attached [42–44]. Accelerometer measurements have been validated for multiple imaging applications, including reconstruction of volumetric images from multiple 2D B-mode slices [43] and analysis of ultrasound operator skill [44].

A limitation of the present study was placement of the ultrasound transducer after the participants had inserted the bolus into their mouths and just before participants were prompted to swallow. This procedure was instituted to minimize interference with patients’ standard-of-care MBSS assessment. It is possible that, because the bolus was often already present in the oral cavity, ultrasound measurements missed movements of the hyoid from its rest position to accommodate the bolus [18, 24]. This limitation could be addressed in a larger study performed outside the standard of care, with boluses administered after the transducer is placed. Transducer placement also contributed to another limitation of the study, the number of swallows that did not meet the criteria for inclusion. Of the 84 paired ultrasound-videofluoroscopy sequences that were successfully synchronized, 28 sequences (one third) were excluded, either because the hyoid bone was out of frame for portions of the ultrasound sequence (26 swallows) or because the hyoid was imaged with resolution insufficient for semi-automatic tracking (2 swallows). More consistent imaging of the hyoid bone by ultrasound is likely feasible in the absence of simultaneous videofluoroscopic imaging, where an ultrasound examiner has more time to confirm proper transducer placement and fewer constraints such as room setup, radiologic imaging equipment, and radiation minimization concerns.

It is worth considering how the methodology employed here for hyoid measurements can be extended to track other relevant swallowing movements, such as those of the tongue, pharynx, and bolus. For example, ultrasound tracking of movements by the tongue root, dorsum, and blade has been shown to be useful for analysis of speech production [45]. Movement of the tongue is a key element of efficient swallowing and extension of this methodology may be useful for analysis of the tongue during swallowing. Similarly, the potential for ultrasound bolus tracking can be demonstrated from the bolus’s visualization in Fig. 1. Imaging of the pharynx using handheld ultrasound has been shown capable to image clinically relevant structures such as the tongue root, palate, vocal cords, and epiglottis [46] and has also been validated for imaging pharyngeal airway dimensions, correlating well with acoustic pharyngometry [47]. However, ultrasound imaging of these structures during swallowing poses additional challenges, beyond the scope of this study. The bolus itself interferes with the strong echoes useful for tongue tracking as it moves along the surface of the tongue, and can be difficult to track throughout a swallow due to its inconsistent boundaries, particularly as it journeys through the hyoid shadow. Pharyngeal tracking, while possible on ultrasound [47], may require transverse rather than sagittal transducer positioning, and would best be validated using an alternative approach such as FEES [48] or manometry [49], instead of videofluoroscopy which images sagittal projections of the vocal tract. Future work should examine whether tracking these additional movements is reliable when compared to videofluoroscopy or FEES.

Validation of handheld ultrasound for measurements of swallowing movements should facilitate additional studies outside of the fluoroscopy suite. Research on characterization of swallowing techniques and patterns has been limited to date, in part to the difficulty of recruiting large normally swallowing populations for videofluoroscopy to establish typical swallowing behaviors and their statistics, given the high variability in normal and abnormal swallows including functional differences in bolus size, consistency (e.g., thin, nectar, honey, pudding), and swallow tasks (e.g., cup sip, sequential). One recent study has reported statistics of normal hyoid motion measured by ultrasound during swallowing of water boluses by 41 healthy participants; rather than handheld ultrasound, this study employed a head stabilization apparatus that likely increased the precision of ultrasound measurements, but could conceivably have altered swallowing behavior [19]. Accurate tracking of hyoid motion using handheld ultrasound, incorporating motion correction like the dynamic translation/rotation method introduced here, could also enable longitudinal studies on larger populations, leading to clinical applications in monitoring the progress of dysphagia and its treatment, in combination with current standard diagnostic approaches. Recent advances in wearable ultrasound arrays [50] may further extend this potential by reducing operator-dependent array motion and interference with swallowing. Wearable arrays may be particularly useful for longitudinally monitoring therapeutic progress and for identifying patients who require imaging-based swallowing assessments (e.g., MBSS or FEES) to fully evaluate changes in function.

Accurate tracking of the hyoid, tongue, and other relevant structures could also provide a new tool for biofeedback during therapeutic maneuvers such as the Mendelsohn maneuver [51], Masako maneuver [52], and effortful swallow [53], potentially improving their efficacy and enabling improved motor learning of swallowing-related movements. Submental ultrasound has already proven useful as a biofeedback tool within speech language pathology, such as in treatment of residual speech sound disorders involving American English /r/[54–56].

## Conclusions

This study has demonstrated that motion of the hyoid can be accurately measured during swallowing using handheld ultrasound with correction for transducer motion. Time-dependent position of the hyoid has been measured in participants with and without aspiration, with good reliability (e.g., 0.75 < ICC < 0.9 for absolute agreement of time-dependent hyoid displacement vs. MBSS measurements) when using motion correction incorporating dynamic translation and rotation. Measured results were consistent with previous measurements for hyoid peak velocity and overall excursion, including trends for the dependence of hyoid velocity on bolus consistency. Validated ultrasound measurement methods may facilitate future studies comparing movements of multiple anatomic structures, enabling further development of clinical applications for swallowing diagnosis and treatment.

## Data Availability

Due to University of Cincinnati legal considerations, access, distribution, and reuse of the raw data supporting results of this study will be limited to uses under a formal Data Use and Sharing Agreement (DUA). Raw data, including simultaneous MBSS and ultrasound video recordings (MP4 format) and data files of tracked swallowing movements (MATLAB format), will be shared upon request, following appropriate execution a DUA by both institutions.
